# The YAP/TEAD Axis as a New Therapeutic Target in Osteosarcoma: Effect of Verteporfin and CA3 on Primary Tumor Growth

**DOI:** 10.3390/cancers12123847

**Published:** 2020-12-20

**Authors:** Sarah Morice, Mathilde Mullard, Regis Brion, Maryne Dupuy, Sarah Renault, Robel Tesfaye, Bénédicte Brounais-Le Royer, Benjamin Ory, Françoise Redini, Franck Verrecchia

**Affiliations:** 1 INSERM UMR1238 “Bone Sarcomas and Remodeling of Calcified Tissues”, Nantes University, F-44035 Nantes, France; sarah.morice@univ-nantes.fr (S.M.); mathilde.mullard@univ-nantes.fr (M.M.); maryne.dupuy@etu.univ-nantes.fr (M.D.); sarah.renault@univ-nantes.fr (S.R.); robel.tesfaye@etu.univ-nantes.fr (R.T.); Benedicte.brounais@univ-nantes.fr (B.B.-L.R.); Benjamin.Ory@univ-nantes.fr (B.O.); francoise.redini@univ-nantes.fr (F.R.); 2 CHU Hôtel Dieu, F-44035 Nantes, France; regis.brion@univ-nantes.fr

**Keywords:** osteosarcoma, Hippo/YAP, tumor growth, verteporfin, CA3

## Abstract

**Simple Summary:**

The low survival rate of osteosarcoma (OS) patients underlines the urgency of developing new therapeutic strategies for this disease. In recent years, the important role of Hippo/YAP signaling in cancer has been evaluated, focusing on the possibility of targeting this signaling pathway as an anti-cancer strategy. The aims of this work were (1) to identify a Hippo/YAP signature in OS patients, (2) to define the role of YAP in OS primary tumor growth, (3) to elucidate the role of TEAD in YAP-driven OS tumor growth in vivo, and (4) to evaluate the effects of verteporfin and CA3, two specific YAP-inhibitors, on the OS tumors growth. Our work identifies the YAP/TEAD axis as a promising therapeutic target in OS and demonstrates that verteporfin and CA3, through regulation of OS cells apoptosis, could be a promising therapeutic strategy for inhibiting OS primary tumor growth.

**Abstract:**

Although some studies suggested that disruption of the Hippo signaling pathway is associated with osteosarcoma progression, the molecular mechanisms by which YAP regulates primary tumor growth is not fully clarified. In addition, the validation of YAP as a therapeutic target through the use of inhibitors in a preclinical model must be demonstrated. RNA-seq analysis and Kaplan–Meier assays identified a YAP signature in osteosarcoma patients and a correlation with patients’ outcomes. Molecular and cellular analysis (RNAseq, PLA, immunoprecipitation, promoter/specific gene, proliferation, cell cycle assays) using overexpression of mutated forms of YAP able or unable to interact with TEAD, indicate that TEAD is crucial for YAP-driven cell proliferation and in vivo tumor growth. In addition, in vivo experiments using an orthotopic mice model of osteosarcoma show that two YAP/TEAD inhibitors, verteporfin and CA3, reduce primary tumor growth. In this context, in vitro experiments demonstrate that these inhibitors decrease YAP expression, YAP/TEAD transcriptional activity and cell viability mainly by their ability to induce cell apoptosis. We thus demonstrate that the YAP/TEAD signaling axis is a central actor in mediating primary tumor growth of osteosarcoma, and that the use of YAP inhibitors may be a promising therapeutic strategy against osteosarcoma tumor growth.

## 1. Introduction

Osteosarcoma (OS) is the most common primary malignant bone tumor identified in children and adolescents [1,2,3]. Although some predisposing genetic factors such as p53 mutations have been discovered, the precise etiology of this pediatric disease remains undetermined [1,4]. The standard treatment of OS is complete surgical resection combined with neo-adjuvant and adjuvant chemotherapies [5,6,7,8]. Unfortunately, OS is a particularly chemotherapy-resistant tumor [9] and resistance to treatment remains one of the leading causes of death in OS patients, with a 5-year survival rate of only around 20–25% [7,10,11]. The lack of response to conventional treatments underlines the urgency of developing new therapeutic strategies. In this context, the progress made in the understanding of the molecular basis of OS pathogenesis in parallel with the emergence of strategies to specifically block signaling pathways associated with cancer progression, seems to be of great interest.

The Hippo signaling pathway plays a key role in the regulation of many cellular processes involved in cancer development, such as cell proliferation, apoptosis, and migration [12,13]. The main intracellular effectors of this signaling cascade are mammalian Ste20-like kinases 1/2 (MST1/2), large tumor suppressor 1/2 (LATS1/2), yes-associated protein (YAP) and its paralog transcriptional coactivator with PDZ-binding motif (TAZ). When this signaling cascade is activated MST1/2 proteins phosphorylate and activate LATS1/2 factors which in turn phosphorylate YAP/TAZ factors, resulting in the inhibition of YAP/TAZ transcriptional activity. When this signaling cascade is not activated, YAP or TAZ are not phosphorylated, and they thus translocate into the nucleus, where they act as co-transcriptional factors [14]. The primary DNA-binding partners of YAP are TEA-domain DNA-binding transcription factor 1–4 (TEAD1–4) able to regulate the expression of genes able to control essential process such as cell proliferation, differentiation and apoptosis [15]. There is growing evidence that disruption of the Hippo signaling pathway or abnormal YAP/TAZ activation is associated with cancers development [13,16,17,18,19,20]. YAP/TAZ have thus been shown to be essential at different stages of cancer progression, such as initiation, progression and metastasis, and for the formation of lung, prostate, breast, liver, stomach, pancreas and brain tumors [21].

Concerning primary bone tumors, tissue array analyses have demonstrated a high level of YAP protein expression in OS tumor tissues compared to surrounding non-cancerous ones [22] and this YAP overexpression is associated to poor prognosis [23]. The molecular mechanisms underlying YAP overexpression in OS seem to be complex, but evidence suggests that it could be due in large part to the stem cell transcription factor SOX2 [24]. The findings of studies using knockdown approaches suggest that YAP and its transcriptional factor TEAD1 participate in the control of OS cell lines proliferation [25]. However, the molecular mechanisms by which YAP regulate tumor growth and the effects of YAP inhibitors, such as verteporfin and CA3, on in vivo OS tumor growth remain to be elucidated. In this context, using molecular and cellular approaches, the aims of this work were (i) to define the role of YAP in OS primary tumor growth, (ii) to elucidated the role of TEAD in YAP-driven OS tumor growth in vivo, and (iii) to evaluate the effects of two specific YAP-inhibitors, verteporfin and CA3, on the OS tumors growth using an orthotopic mouse model.

## 2. Results

### 2.1. The Elevation of Hippo Target Genes Expression in OS Patients Is Associated with the Overall Survival of Patients

**Figure 1:**
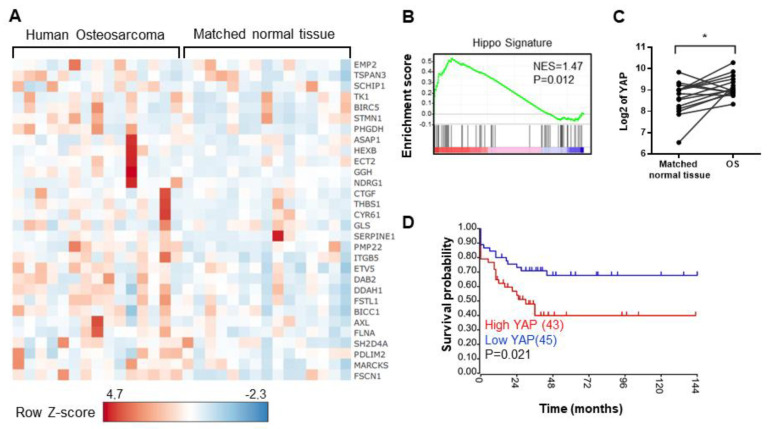
Elevation of Hippo gene expression in osteosarcoma (OS) patients—correlation between YAP expression and overall survival of OS patients. (**A**) Heatmap showing color-coded expression of Hippo target genes in OS tissue and matched normal tissue from the same OS patient following bioinformatics analysis of RNAseq data GSE99671 [26] from a cohort of 15 OS patients. High expression (red), low expression (blue). (**B**) GSEA showing a Hippo signature in OS samples following bioinformatics analysis of RNAseq data GSE99671 [26] from an OS patient’s cohort comprising 15 samples. FDR false discovery rate, NES normalized enrichment score. (**C**) Relative YAP gene expression in OS samples and matched normal tissues of the same patient following bioinformatics analysis of RNAseq data GSE99671 [26] (* *p* < 0.05). (**D**) Kaplan–Meier analysis of the survival outcome of patients dichotomized into high and low YAP levels, following analysis of the RNAseq dataset GSE42352 [27] from an OS patient cohort comprising 88 samples. Analysis was performed using R2 (http://r2.amc.nl); *p*-value is from log-rank tests.

To resolve outstanding questions regarding the role of the Hippo signaling pathway in OS development, we attempted to identify for a hypothetical Hippo signature in OS patients, using publicly available databases. Gene set enrichment analysis (GSEA) of expression data obtained by using RNAseq assays from a cohort of OS patients reveals a Hippo conserved signature in OS samples as compared to normal bone samples from the same patient (Figure 1A, human osteosarcoma vs. matched normal tissue, and Figure 1B), suggesting a hyperactivity of the Hippo signaling pathway in OS patients. Indeed, multiple Hippo-regulated genes are significantly overexpressed in OS compared with normal bone tissue. These genes include, for example, CYR61, THBS1, PAI-1, and BIRC5, previously described as YAP target genes in tumor tissues (Figure 1A and Figure S1). Consistently, YAP is overexpressed in OS samples compared with normal tissue from the same patient (Figure 1C). Interestingly, high YAP transcripts significantly correlate with poor survival outcome in OS patients as illustrated in the Kaplan-Meier plot in Figure 1D.

Taken together, these results highlight that the Hippo/YAP signature correlates with a poor survival outcome in OS patients.

### 2.2. YAP/TEAD Interactions Are Crucial to Promote YAP-Driven TEAD Transcriptional Activity in OS Cells

**Figure 2:**
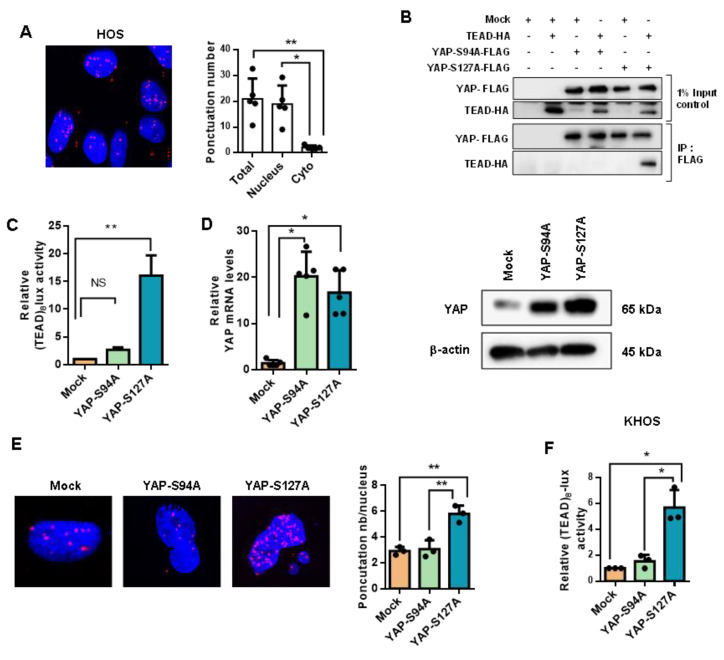
Role of TEAD in YAP-driven transcriptional activity. (**A**) Localization of endogenous YAP/TEAD1 complexes by in situ PLA in HOS cells. The red signal was obtained using Alexa555-labeled hybridization oligo nucleotides targeting amplified in situ PLA products. DAPI (blue) staining was used for nuclear visualization (**left panel**). Bars indicate means ± SD of three independent experiments (* *p* < 0.05, ** *p* < 0.01, **right panel**). (**B** HEK293 were transiently co-transfected with the YAP-S94A, YAP-S127A, TEAD1 or empty vector as indicated. 48 h after transfection lysates were subjected to immunoprecipitation (IP) with anti-Flag antibody followed by Western blotting (WB) by anti-Flag and anti-HA antibodies as indicated. (**C**) HOS cells were co-transfected with the TEAD-specific construct (TEAD)8-lux with or without empty, YAPS94A and YAPS127A expression vectors. Bars indicate means ± SD of four independent experiments, each performed in triplicate (** *p* < 0.01). (**D**) YAP mRNA steady-state levels were quantified by RT-q-PCR analysis in mock-, YAPS94A- and YAPS127A-transfected K-HOS cells. Bars indicate means ± SD of four independent experiments, each performed in duplicate (* *p* < 0.05, **left panel**). YAP production was detected by Western blot analysis in mock-, YAPS94A- and YAPS127A-transfected K-HOS cells. Results shown are representative of two independent experiments (**right panel**). (**E**) Localization of YAP/TEAD1 complexes by in situ PLA experiments in mock-, YAPS94A- and YAPS127A-transfected K-HOS cells. The red signal was obtained using Alexa555-labeled hybridization oligo nucleotides targeting amplified in situ PLA products. DAPI (blue) staining was used for nuclear visualization (**left panel**). Bars indicate means ± S.D. of three independent experiments (** *p* < 0.01, **right panel**). (**F**) Mock-, YAPS94A- and YAPS127A-transfected cells were transiently transfected with the TEAD-specific construct (TEAD)8-lux. Bars indicate means ± SD of four independent experiments, each performed in duplicate (* *p* < 0.05).

Since some previous studies indicated that activation of the Hippo/YAP signaling pathway induces tumor progression through the recruitment of YAP to DNA by the TEAD transcription factor family, we began our analysis by examining the relationship between YAP and TEAD in a panel of three human OS cell lines: HOS, MG63 and G292 cells. In situ PLA assays clearly demonstrate that YAP and TEAD interact (proteins localized within 40 nm of each other) in the nucleus of OS cell lines (Figure 2A and Figure S2A). To elucidate the role of TEAD in YAP-driven OS development, we then probed the consequences of YAP activation able or unable to interact with TEAD, using overexpression of either YAPS127A (constitutively active, TEAD-binding YAP protein) or YAPS94A (TEAD-binding deficient YAP protein). We first verified the expression and functionality of these mutated YAP expression vectors in transient transfection assays. As anticipated, unlike YAPS127A, YAPS94A proteins are unable to bind TEAD1 (Figure 2B) and do not induce a transcriptional response in OS cells (Figure 2C and Figure S2B). Using retroviral infection, we then established K-HOS clones stably overexpressing YAPS127A, YAPS94A or empty vector. As shown in Figure 2D, YAPS94A- and YAPS127A-transfected cells express high levels of YAP mRNA (left panel) and YAP protein (right panel). In situ PLA assays demonstrate increased YAP-TEAD interactions in the nucleus of K-HOS cells in YAPS127A cells compared to YAPS94A- or mock-transfected cells (Figure 2E). In addition, unlike YAPS94A- and mock-transfected cells, YAPS127A cells exhibit an increased-TEAD transcriptional response as measured by luciferase reporter gene assay with the TEAD-specific reporter construct (TEAD)8-lux (Figure 2F).

Taken together these results highlight that YAP/TEAD interactions are crucial in the ability of YAP to drive transcriptional activity in OS cells.

### 2.3. OS Cell Proliferation and In Vivo OS Tumor Growth Critically Depend on YAP-TEAD Interactions

**Figure 3:**
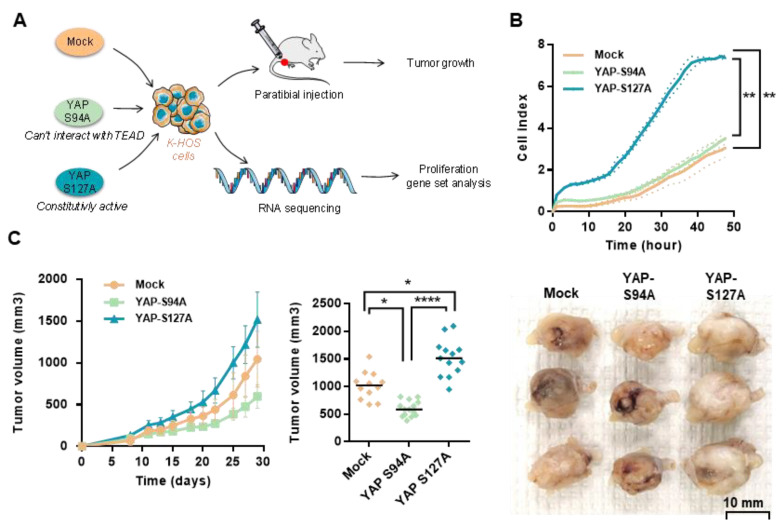
Role of TEAD in YAP-driven OS cell proliferation and in vivo OS tumor growth. (**A**) Schematic representation of the experimental protocols. Briefly, K-HOS cells were stably transfected with mock-, YAPS94A- or YAPS127A-vectors. Intramuscular paratibial injections of these cells were performed in nude mice, and the tumor volume was measured three time per week. In parallel, RNAseq analysis was performed on cells. (**B**) Realtime proliferation assays were performed to compare the cell proliferation rate between mock-, YAPS94A- and YAPS127A-transfected K-HOS. Each point indicates means ± SD of three independent experiments, each performed in sextuplicate (** *p* < 0.01). (**C**) Intramuscular paratibial injections of 1.106 mock-, YAPS94A- and YAPS127A-transfected K-HOS cells were performed in three groups of 12 nude mice. Tumor volumes were measured three times per week for 4 weeks (**left panel**). Means tumor volumes of each group were measured 29 days after cell injection (**middle panel**, mean ± SD; * *p* < 0.05, **** *p* < 0.001). Photographs show three representative bone tumors in each group of mice (**right panel**).

Using these OS cellular tools, we then examined the functional role of TEAD in YAP-driven OS cell proliferation and in vivo tumor growth (Figure 3A). Real-time proliferation assays demonstrate an increase of OS cell proliferation when YAPS127A is overexpressed compared with the ability of OS cells to proliferate when YAPS94A or an empty vector is overexpressed (Figure 3B). A preclinical experimental model of OS induced by orthotopic injection of either YAPS94A-, YAPS127A- or mock-transfected OS cells demonstrates the crucial role of TEAD in YAP-driven in vivo OS tumor growth (Figure 3C). Indeed, 29 days after cell injection, the tumor volume is significantly increased when TEAD-interacting YAP is overexpressed (YAPS127A cells versus mock-transfected cells and YAPS127A cells versus YAPS94A cells) (Figure 3C). In contrast, the tumor volume is significantly reduced when TEAD-binding deficient YAP is overexpressed (YAPS94A- versus mock-transfected cells and YAPS94A- versus YAPS127A-transfected cells). Specifically, the mean tumor size at day 29 was 1043 ± 333 mm^3^ in the control group (mock-transfected cells), 1517 ± 330 mm^3^ when TEAD-interacting YAP is overexpressed (YAPS127A cells), and only 599 ± 140 mm^3^ when TEAD-binding deficient YAP is overexpressed (Figure 3C).

Taken together, these results demonstrate the crucial role of TEAD in YAP-driven cell proliferation and in vivo tumor growth in OS preclinical models.

### 2.4. Role of TEAD in YAP-Driven Cell Cycle Genes Expression

**Figure 4:**
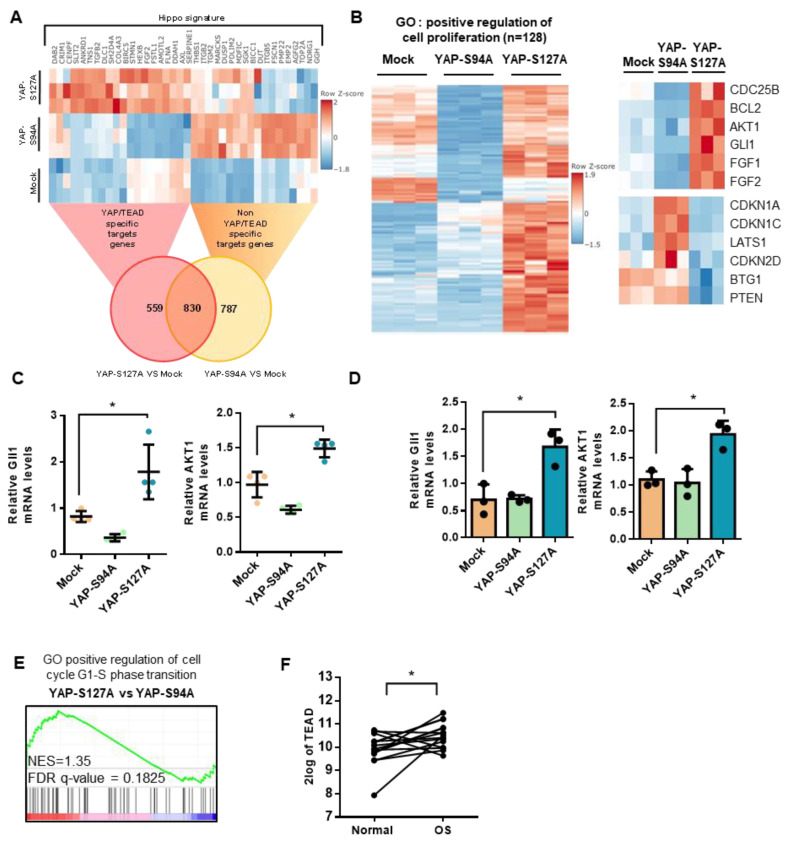
Role of TEAD in YAP-driven cell cycle genes expression. (**A**) Heat map of YAP-associated upregulated gene signature in mock-, YAPS94A- and YAPS127A-transfected K-HOS cells. Color scales are based on Z-scores. (**B**) Heat map showing mRNA levels of 128 genes significantly overexpressed in YAPS127A-transfected cells involved in the positive regulation of cell proliferation. Color scales are based on Z-scores. (**C**) Total RNA was extracted from tumor biopsies of mice injected with mock-, YAPS94A- or YAPS127A-transfected cells. Gli1 and AKT1 mRNA steady-state levels were determined by quantitative RT-PCR. Bars indicate means ± S.D. of three independent experiments, each performed in duplicate. (* *p* < 0.05). (**D**) Gli1 and AKT1 mRNA steady-state levels were quantified by RT-qPCR analysis in mock-, YAPS94A- and YAPS127A-transfected K-HOS cells. Bars indicate means ± SD of three independent experiments, each performed in triplicate (* *p* < 0.05). (**E**) Enrichment score (ES) plots of GSEA analysis show a significant upregulation of genes involved in G1-S phase transition in YAPS127A-transfected K-HOS cells compared to YAPS94A-transfected K-HOS cells. (**F**) Relative TEAD1 gene expression in OS patients and control samples of the same patients following bioinformatics analysis of RNAseq data GSE99671 [26]. From an OS patient cohort comprising 15 samples. (* *p* < 0.05).

To gain more insights into the crucial role of TEAD in YAP-driven cell proliferation and tumor growth, we then compared the RNA sequencing transcriptional profiles of YAPS127A-, YAPS94A- and mock-transfected cells. As shown in Figure S3, mock-, YAPS94A- and YAPS127A-transfected cells display distinct transcriptional profiles, with multiple genes significantly differentially expressed. Transcriptional analysis thus identifies 1617 genes whose expression is regulated by the overexpression of the YAP mutated proteins able to bind TEAD (YAPS127A) or not (YAPS94A). Of these, 559 genes require the interaction between YAP and TEAD (Figure 4A). RNA-seq analysis identifies 128 genes related to positive regulation of cell proliferation (Figure 4B) that are significantly overexpressed in YAPS127A cells (compared to mock- or YAPS94A-transfected cells). These include genes directly involved in the control of cell cycle, such as CDC25B, and cell proliferation, such as Gli1 or AKT (Figure 4B, right panel). In contrast, in YAPS94A cells, the expression of some genes related to inhibition of cell proliferation is increased, such as CDKN1A, CDKN1C, CDKN2D and LATS1 (Figure 4B, right panel). Interestingly, quantitative PCR analysis indicates that the expression of gli1 and AKT genes by tumor cells from mice biopsies and from cultured cells are upregulated when YAP able to interact with TEAD is over-expressed (Figure 4C,D). In addition, GSEA analysis indicates that overexpression of YAP-S127A increase the expression of genes involved on positive regulation of cell cycle G1-S phase transition (Figure 4E). This strongly demonstrates the crucial role of TEAD in YAP-driven gene expression, which is implicated in the regulation of both OS cell proliferation and in vivo tumor growth. Finally, to investigate the clinical importance of the role played by TEAD in OS tumor development, we analyzed TEAD gene expression using data extracted from the GSE99671 database [26]. Analysis of OS RNAseq data demonstrates that TEAD is overexpressed in OS biopsies compared with control samples from the same patient (Figure 4F).

Taken together, these results demonstrate: (a) the crucial role of TEAD in YAP-driven cell cycle genes expression, and (b) that TEAD is overexpressed in OS samples compared with normal tissue from the same patient.

### 2.5. Verteporfin and CA3 Inhibit OS Primary Bone Tumor

To validate YAP/TEAD signaling as a potential therapeutic target for OS treatment, we evaluated the effect of verteporfin and CA3, two Hippo/YAP inhibitors, on primary tumor growth in a preclinical model of OS.

**Figure 5:**
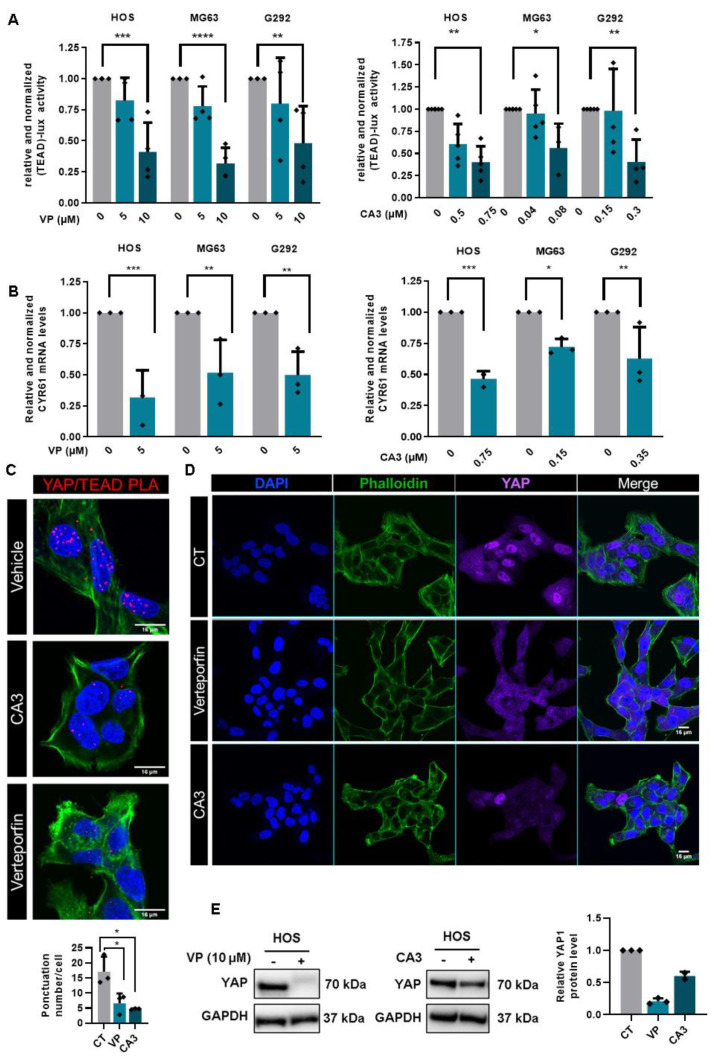
Verteporfin and CA3 inhibit YAP expression and YAP-driven TEAD transcriptional activity. (**A**) HOS, MG63 and G292 cells were transfected with the TEAD-specific construct (TEAD)8-lux. 24 h after transfection, cells were treated with verteporfin (left panel) or CA3 (right panel) as indicated concentration for 48 h. Bars indicate means ± S.D. of at least three independent experiments, each performed in duplicate (* *p* < 0.05, ** *p* < 0.01, *** *p* < 0.001, **** *p* < 0.0001). (**B**) CYR61 mRNA steady-state levels were quantified by RT-q-PCR analysis in the presence or absence of verteporfin (left panel) or CA3 (right panel) 48 h. Bars indicate the means ± SD of three independent experiments, each performed in duplicate (* *p* < 0.05, ** *p* < 0.01, *** *p* < 0.001,). (**C**) Localization of YAP/TEAD1 complexes by in situ PLA experiments in HOS cells treated or not with 10 µM verteporfin and 0.75 µM CA3 during 48 h. The red signal was obtained using Alexa555-labeled hybridization oligo nucleotides targeting amplified in situ PLA products. DAPI (blue) staining was used for nuclear visualization (**left panel**). Bars indicate means ± S.D. of three independent experiments (*p < 0.05, **right panel**). (**D**) HOS were stimulate or not with 10 µM verteporfin and 0.75 µM CA3 during 48 h and were then fixed, permeabilized and stained with a monoclonal antibody directed against YAP (far-red). F-actin cytoskeleton and nuclei were respectively revealed by phalloidine (green) and DAPI labelling (blue). Photographs representative of two independent experiments are shown. (**E**) YAP production was detected by Western blot analysis in HOS cells treated or not with 10 µM verteporfin and 0.6 µM CA3 during 72 h. Results shown are representative of three independent experiments (right panel).

We first validated that verteporfin and CA3 block the YAP/TEAD cascade, as they inhibit transactivation of the TEAD-specific reporter construct (TEAD)8-lux (Figure 5A) and the expression of CYR61 (Figure 5B) or CTGF (Figure S4A) two target genes of the YAP/TEAD cascade. Note that neither verteporfin nor CA3 inhibits transactivation of the AP-1- and NFkB-specific reporter construct (Figure S4B). In situ PLA assays clearly demonstrate that YAP and TEAD interactions is significantly reduced when the cells are treated with verteporfin or CA3 (Figure 5C and Figure S4C). To elucidate the mechanism underlying the effect of verteporfin and CA3 on YAP/TEAD transcriptional activity, we then evaluated the expression of YAP by immunofluorescence. A shown in Figure 5D and Figure S4D, verteporfin and CA3 reduce the expression of YAP. These results, confirmed by Western-blot analysis (Figure 5E and Figure S4E) suggest that verteporfin and CA3 reduce TEAD transcriptional activity mainly by their ability to reduce YAP expression and thus YAP/TEAD interaction. In addition, an experience suggests that TEAD production is also affected by verteporfin and CA3 (Figure S4F).

**Figure 6:**
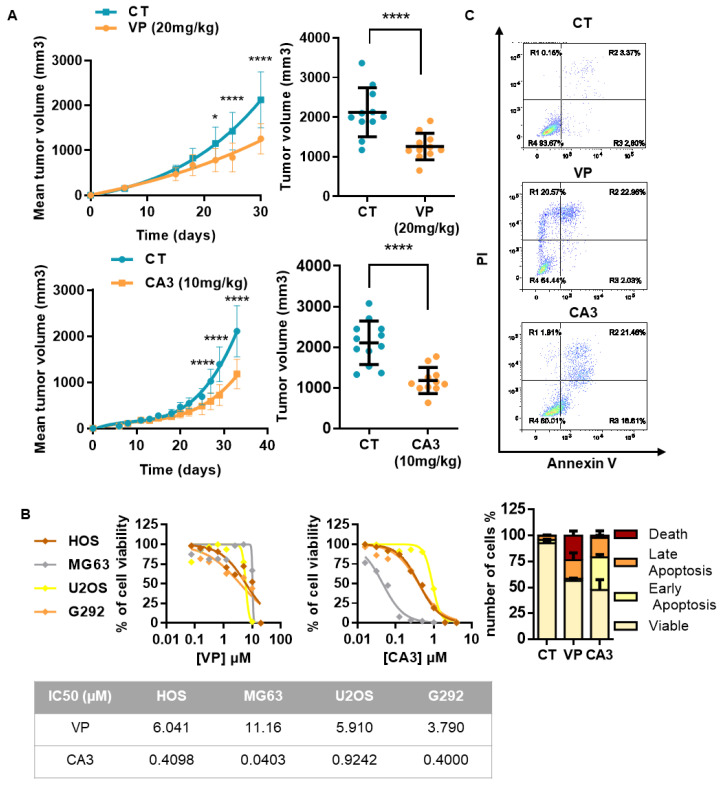
Verteporfin and CA3 inhibits OS primary bone tumor growth. (**A**) Intramuscular paratibial injections of 1.106 HOS cells were performed in two groups of 12 nude mice treated with vehicle or verteporfin (20 mg/kg), and vehicle or CA3 (10 mg/kg) as indicated. Tumor volumes were measured three times per week for 4 weeks. Means tumor volumes of each group were measured 30 or 33 days after cell injection (right panels, mean ± SD; * *p* < 0.05 **** *p* < 0.0001). (**B**) HOS, MG63, and G292 OS cell lines were treated with verteporfin (left panel) or CA3 (right panel) as indicated for 72 h. Graph represent cell viability after treatment. Mean of three independent experiments, each performed in sextuplicate. (**C**) Upper panels: Representative dot plots of HOS cells untreated or treated with 10 µM verteporfin or 0.75 µM CA3 for 72 h are shown (representative graphs of three experiments). Lower panels: Bars indicate the means ± SD of the relative number of lives cells, death cells, and cells in early- or late-phase apoptosis (n = 3 independent experiments).

Importantly, experiments using an orthotopic preclinical model of OS demonstrate that injection of verteporfin or CA3 inhibit the OS tumor growth in vivo (Figure 6A). Indeed, respectively 30 and 33 days after tumor cell injection, the bone tumor volume is significantly decreased in mice treated with verteporfin or CA3. Regarding verteporfin experiments, the mean tumor size at day 30 was 2122 ± 618 mm^3^ when the mice were treated with vehicle (control group) and only 1258 ± 334 mm^3^ when the mice were treated with 20 mg/kg of verteporfin (Figure 6A, right and upper panel). Regarding CA3 experiments, the mean tumor size at day 33 was 2123 ± 535 mm^3^ when the mice were treated with vehicle (control group) and only 1179 ± 319 mm^3^ when the mice were treated with 10 mg/kg of CA3 (Figure 6A, right and lower panel). In vitro assays demonstrate that verteporfin and CA3 affect the cell viability of the three OS cell lines used; HOS, G292, and MG63, in a dose-dependent manner (Figure 6B). Flow cytometric Annexin V/PI assay showed that verteporfin and CA3 induce early and late apoptotic events, and cell death (Figure 6C and Figure S5). For example, the percentage of HOS cells in early apoptosis (Annexin V+/PI−) was 3.2 ± 0.6% in the absence of drug, and was 2.1 ± 0.1% and 31.6 ± 2.8% after 72 h treatment of cells with verteporfin and CA3, respectively. The percentage of HOS cells in late apoptosis (Annexin V+/PI−) was 3.9 ± 0.7% in the absence of drug, and reached 17.9 ± 7.1% and 18.7 ± 6.9% after 72 h treatment of cells with verteporfin and CA3, respectively. The percentage of HOS death cells was 0.2 ± 0.1% in the absence of drug, and reached 23.7 ± 4.4% and 2.3 ± 0.3% after 72 h treatment of cells with verteporfin and CA3, respectively.

Together, these results demonstrate that verteporfin and CA3 (i) inhibit TEAD transcriptional activity mainly via their ability to reduce YAP expression and thus YAP/TEAD interactions, (ii) inhibit in vitro OS cell lines viability, (iii) reduce in vivo primary tumor growth and (iv) suggest that these later effects are mainly due to their ability to induce cell apoptosis.

## 3. Discussion

The lack of response to drugs is a major challenge to define effective treatment in OS. Although chemotherapy significantly improved the prognosis of OS patients after the development of neoadjuvant therapy in the early 1980s [28], the results have not improved since then and are approximately 70% for 5-year survival. The remaining 30% of patients are resistant to several types of chemotherapy. In this context it seems essential to develop new approaches to improve survival.

### 3.1. YAP/TEAD Signaling as a Target Therapy against Primary Tumor Growth

High YAP expression and/or YAP activation have been described in several solid tumor types and correlated with poor prognosis [19]. It has been proposed that YAP acts as an oncogene through activation of target genes that especially promote stimulation of tumor cell proliferation [29,30]. Despite the emerging importance of YAP in many cancers, the exact mechanisms driving key functions in cancer progression still remain to be resolved. While YAP expression was reported in OS, the molecular mechanisms underlying primary tumor growth have not been established in this pathology.

In this work, we first demonstrate that YAP is highly expressed in biopsies from OS patients and confirm a previous study reporting that YAP expression predicts a poor prognosis in this pathology [23]. We demonstrate the crucial role of YAP in the control of OS cell proliferation and tumor growth. Indeed, the overexpression of a constitutively active YAP (YAPS127A) promotes both the in vitro proliferation of OS cells and the in vivo growth of primary bone tumors. In several cancers, it has been demonstrated that YAP stimulates cell proliferation largely by controlling the expression of a broad number of cell cycle regulators or the expression of oncogenes, for example MYC and AP-1 family members [19]. In this work, we identify genes directly involved in the control of cell cycle, such as CDC25B, or involved in the regulation of oncogene expression, such as Gli1 previously described as a pro-proliferation factor and thus as a potential therapeutic target in OS [31]. Furthermore, using overexpression of a mutant YAP protein unable to interact with TEAD1–4 (YAPS94A), we clearly demonstrate that the TEADs transcriptional factors are crucial in YAP-driven OS growth both in vitro and in vivo as previously described in other cancers [21]. Reinforcing these results, TEAD1 has been found to be highly expressed in OS patients. Together with the previous observation that TEAD1 plays a crucial role in the regulation of OS cell proliferation [25], these results strongly support the hypothesis that the YAP/TEAD axis could represent a promising target to inhibit primary OS tumor growth. Regarding the main role of TEAD transcriptional factor in the expression of YAP-driven genes, we further performed transcriptomic analysis in OS cells that identified TEAD-independent and TEAD-dependent modulation of YAP target genes in OS.

### 3.2. Suppression of Primary Tumor Growth by YAP/TEAD Inhibitors

To validate YAP/TEAD axis as a potential therapeutic target in OS, we evaluated the effect of two YAP inhibitors, verteporfin and CA3, on OS tumor growth [32]. Verteporfin is a light-activated drug used in photodynamic therapy for the treatment of choroidal neovascular membranes [33]. CA3 is a novel YAP inhibitor recently selected and identified through chemical library screening [34]. We specifically demonstrate that verteporfin and CA3 inhibit primary OS tumor growth. In this context, we show that verteporfin and CA3 block in vitro cell proliferation and induce in vitro cell apoptosis. In accordance with our results, verteporfin was subsequently reported to inhibit the growth of malignant cells without light activation, such as in human glioma [35]. CA3 was seen to strongly inhibits esophageal adenocarcinoma cell growth in vitro and exerts antitumor activity in xenograft model [34]. Both inhibitors suppress mesothelioma cancer stem cell phenotype and tumor formation [36]. Initially described as a YAP/TEAD interaction inhibitor [33], verteporfin was recently described as able to induce the degradation of YAP protein, demonstrating its capacity to target the YAP cascade via different modes of action [36,37]. Here, we demonstrate that verteporfin reduces YAP expression and thus YAP-driven TEAD transcriptional activity in OS cell lines. We cannot exclude that verteporfin also inhibit YAP/TEAD direct interaction and therefore YAP/TEAD transcriptional response [38]. Regarding CA3, as previously described in only one recent work using mesothelioma cells, we demonstrated that CA3 induce the decrease of YAP production in OS cell lines [36]. Whatever the mechanisms of action, our work clearly shows that treatment with verteporfin or CA3 reduce YAP/TEAD signaling in OS cells as demonstrated using specific TEAD promoter/gene reporter assays. In addition, we have shown that verteporfin and CA3 induce cell death by apoptosis in the two cell lines tested, and therefore induce OS cell death and thus inhibits the tumor growth in vivo. These finding are in accordance with previous observations using cultured tumor cells in that verteporfin or CA3 induce apoptosis of tumor cells [36,39]. Notably, although we have shown that verteporfin and CA3 affect the YAP/TEAD signaling pathway in osteosarcoma, we cannot totally exclude that some of the effects of these drugs on cell death and tumor growth are associated with effects independent of the YAP/TEAD pathway.

## 4. Materials and Methods

### 4.1. Osteosarcoma Mouse Model

Four-week-old female Rj:NMRI-nude mice (Janvier, Le Genest Saint Isle, France) were maintained under pathogen-free conditions at the Experimental Therapy Unit (Faculty of Medicine, Nantes, France) in accordance with the institutional guidelines of the French Ethical Committee (CEEA Pays de la Loire n°06: project authorization 8405). Mice received an intramuscular injection of 1.10^6^ K-HOS parental or mutant cells in close proximity to the tibia. Five days after cell injections, groups of mice received or not the molecules verteporfin (20 mg/kg), CA3 (10 mg/kg) or control vehicle twice a week by intraperitoneal injection. The tumor volume (V) was calculated from the measurement of three perpendicular diameters using a caliper according to the following formula: V = (length × height × depth)/2.

### 4.2. Cell Culture and Reagents

K-HOS (CRL-1544), HOS-MNNG (CRL-1543), MG-63 (CRL-1427) and G292 (CRL-1423) osteosarcoma cell lines were purchased from ATCC (LGC Standards, Molsheim, France). HEK293 cells were purchased from Invitrogen (Thermo Fisher, Courtaboeuf, France). Cells were cultured in DMEM (Dulbecco’s Modified Eagle’s Medium, Lonza, Basel, Switzerland) supplemented with 10% fetal calf serum (Hyclone Perbio, Bezons, France).

To generate mutant YAP-S94A and YAP-S127A expressing cells, retrovirus infection was performed by transfecting 293 Phoenix retrovirus packaging cells with empty vector, pQCXIH-Myc-YAP-S94A and pQCXIH-Flag-YAP-S127A. pQCXIH-Myc-YAP-S94A and pQCXIH-Flag-YAP-S127A were gifts from Kunliang Guan (respectively Addgene plasmid #33094 and #33092; http://n2t.net/addgene:33094 and 33092; RRID:Addgene-33094 and -33092). pQCXIH CMV/TO DEST (w382-1) was a gift from Eric Campeau and Paul Kaufman (Addgene plasmid #17394; http://n2t.net/addgene:17394; RRID:Addgene-17394). 24 h post-transfection, retroviral supernatant was supplemented with 5 µg/mL polybrene (Santa Cruz Biotechnology, CA, USA), filtered through a 0.45-µm filter and used to infect K-HOS cells. 48 h after infection, cells were selected with 200 µg/mL hygromycin-B (Invitrogen, Courtaboeuf, France). Verteporfin and CA3 were respectively purchased from Tocris (Bristol, UK), and InvivoChem (Libertyville, IL, USA).

### 4.3. Luciferase Reporter Assay and Plasmid Constructs

Transient cell transfections were performed with jetPEI™ (Polyplus transfection, Illkirch, France). The pRLTK-Renilla luciferase expression vector was co-transfected in all experiments to monitor transfection efficiencies. Luciferase activity was determined with the Dual-Luciferase reporter assay system (Promega, Charbonnieres, France). The 8xGTIIC-Luc (gift from Stefano Piccolo, Addgene plasmid #34615; http://n2t.net/addgene:34615; RRID:Addgene-34615, (TEAD)8-lux in the text) construct was used as a specific reporter construct specific for TEAD-driven signaling.

### 4.4. Real Time Proliferation and Annexin V Assays

In vitro cell proliferation assays were assessed using xCELLigence Real-Time cell-Analyzer (ACEA Biosciences, San Diego, CA). The experiment was done in triplicate and repeated three times.

Annexin V assay: OS cells were cultured and treated with or without verteporfin or CA3 for 72h. Cells undergoing apoptosis were identified by flow cytometry (Fortessa, BD Biociences, San Jose, CA) using the FITC Annexin V Apoptosis Detection Kit I (BD Biociences).

Osteosarcoma cell lines were plated in DMEM with 10% fetal calf serum and treated with Verteporfin and CA3 as indicated. Cell growth and viability were determined by using a WST-1 cell proliferation assay kit (Takara bio Inc., Kusatsu, Japan). Absorbance of the samples was measured at 440 nm wave length with Victor2™ (Perkin Elmer, life sciences) ELISA reader after 2 h incubation.

### 4.5. Immunofluorescence

Cells were seeded onto Ibidi µ-Slide 8 Well overnight and treated with or without verteporfin or CA3 for 48 h, fixed with 4% paraformaldehyde and permeabilized with 0.5% Triton. Samples were incubated with Anti-YAP antibody (Cell signaling Technology, Leiden, The Netherlands). F-actin and nucleus were stained using respectively Alexa-fluor 488 phalloidin and DAPI. Images were acquired using a confocal microscope (NIKON A1 N-SIM) and processed using ImageJ.

### 4.6. RNA Extraction and Real-Time Polymerase Chain Reaction

RNA was extracted from cells and tumors using NucleoSpin^®^RNAplus (Macherey Nagel, Duren, Germany) and reverse transcribed using the Maxima H minus first stand cDNA synthesis kit (Thermo Fisher, Courtaboeuf, France). Real-time monitoring of PCR amplification of complementary DNA was performed using DNA primers (primer sequences are available in Table 1) using QuantStudio 7 Flex Real-Time PCR System (Thermo Fisher) with SYBR^®^ Select Master Mix (Life Technologies, Carlsbad, CA). Target gene expression was normalized to glyceraldehyde 3-phosphatedehydrogenase (GAPDH) and β-actin (ACTB) levels in respective samples as an internal standard.

### 4.7. Western Blot Analysis

Equal amounts of total protein extracts (lysis buffer: SDS 1%, Tris pH 7.4 10mM, Sodium orthovanadate 1 mM). were separated by SDS-polyacrylamide gel electrophoresis (SDS-PAGE) and transferred to PVDF Transfer membrane (Thermo Scientific, Illkirch, France). Antibodies used for western blotting were YAP1 (Proteintech, Manchester, UK), anti-Flag (Sigma Aldrich), HA-tag (Cell signaling), β-actin (Cell signaling), anti-mouse IgG-HRP (Santa Cruz Biotechnology, CA, USA), anti-rabbit IgG-HRP (Santa Cruz). Antibody binding was visualized with the enhanced chemiluminescence system (SuperSignal West Pico Chemiluminescent Substrate, Thermo Scientific, Illkirch, France). For quantification, luminescence was detected with a Charge Couple Device (CCD) camera and analyzed using the GeneTools program (Syngene, Cambridge, UK).The original western blot figures can be found in supplemental materials (Figure S6).

### 4.8. Immunoprecipitation

HEK239FT cells were transfected with different vectors: pPGS-3HA-TEAD1, pCMV-Flag-YAP-S94A and pCMV-Flag-S127A-YAP were gifts from Kunliang Guan (Addgene plasmids #33055, #33102 and #27370; RRID: Addgene-33055, -33102 and -27370).

24 h after transfection, media were changed with fresh DMEM containing 1% FCS for 24 h. Transfected cells were then rinsed with ice-cold PBS and lysed in IP-lysis buffer (Invitrogen, Courtaboeuf, France). Equal amounts of proteins were precleared overnight at 4 °C using Protein-A/G-agarose (Santa Cruz Biotechnology, CA, USA). Supernatants were incubated with primary antibody against Flag (Sigma Aldrich) and HA-tag (Cell signaling), for 2 h at 4 °C. 50 µL of Protein-A/G-agarose was then added and incubated overnight at 4 °C. Beads were washed three times with IP-lysis buffer; thereafter, 30 µL of Laemmli buffer was added and boiled for 5 min. After centrifugation, supernatants were harvested and processed for SDS-PAGE and Western blot as described above.

### 4.9. In Situ Proximity Ligation Assay (PLA), Immunofluorescence and Confocal Microscopy

Duolink PLA ^®^: 5 × 103 OS cells were seeded in Ibidi µ-Slide VI 0.4. 24 later, media was changed to DMEM with 1% FBS. Cells were then fixed with 4% PFA for 15 min at room temperature and incubated overnight at 4 °C with primary antibody against YAP (Cell signaling or Santa Cruz Biotechnology), TEF-1 (Santa Cruz). In situ PLA was performed using DuoLink in Situ Reagents (Sigma-Aldrich) according to the manufacturer’s instructions.

Immunofluorescence assays: cells were seeded onto Ibidi µ-Slide 8 Well overnight, fixed with 4% paraformaldehyde for 15 min and permeabilized with 0.5% Triton. Samples were incubated with Anti-Vinculin−FITC antibody (Sigma-Aldrich). F-actin and nucleus were stained using respectively Alexa-fluor 488 phalloidin and DAPI. Images were acquired using a confocal microscope (NIKON A1 N-SIM) and processed using ImageJ.

### 4.10. RNA-seq Analysis

RNAseq analysis was performed by Active Motif (Carlsbad, CA, USA). Differential gene expression analysis was performed using DESeq2 package. The p-values obtained were corrected for false positives by using Independent Hypothesis Weighting (package IHW) multiple testing adjustment method. Genes were considered significantly differentially expressed if log2 fold-change was over 1 or less than −1 and FDR was less than 0.05. For the differentially expressed genes, over-representation and gene set enrichment analysis (GSEA) were done using clusterProfiler package, and results were plotted using enrichPlot. GSEA was performed using GSEA software (http://software.broadinstitute.org/gsea/). Gene sets used are described in Table 2.

### 4.11. Statistical Analysis

Histogram and data are shown as mean +/− S.D. of a minimum of three independent experiments.

Statistical analyses were performed using GraphPad Prism version 6 for Windows (GraphPad Software, La Jolla, CA, USA), www.graphpad.com. The Wilcoxon matched test was used to compare the expression levels between OS and matched normal tissue. The Mann-Whitney test was used to compare the difference between two groups. A *p*-value under or equal to 0.05 was considered statistically significant.

### 4.12. Database

RNA sequencing data of OS patient and matched normal tissue were downloaded from the Gene Expression Omnibus database (GSE99671, https://www.ncbi.nlm.nih.gov/geo/query/acc.cgi?acc=GSE99671).

Kaplan Meier analysis of osteosarcoma patient tumor samples was performed using the R2 Genomics Analysis and Visualization Platform. Genome-wide gene expression analyses of high-grade osteosarcoma are from (GSE42352 https://www.ncbi.nlm.nih.gov/geo/query/acc.cgi?acc=GSE42352).

## 5. Conclusions

Our data clearly demonstrated that (1) the Hippo/YAP signature correlates with a poor survival outcome in OS patients, (2) the crucial role of TEAD in YAP-driven cell proliferation and in vivo tumor growth in OS, and (3) verteporfin and CA3, two YAP/TEAD transcriptional inhibitors, significantly reduce in vivo primary tumor growth mainly due to their ability to induce cell apoptosis. In this context, this work forms the basis for the development of better approaches to improve the survival of osteosarcoma patients by identifying the YAP/TEAD axis as a promising therapeutic target. In addition, we demonstrated that YAP/TEAD transcriptional inhibitors, such as verteporfin and CA3, represent promising therapeutic drugs in OS.

## Figures and Tables

**Table 1:** Primer sequences for quantitative RT-PCR.

Gene	Forward	Reverse
AKT1	TACGAGAAGAAGCTCAGCCC	TTGGTCAGGTGGTGTGATGG
Gli1	CCAACTCCACAGGCATACAGGAT	CACAGATTCAGGCTCACGCTTC
GAPDH	TGGGTGTGAACCATGAGAAGTATG	GGTGCAGGAGGCATTGCT
YAP1	TGACCCTCGTTTTGCCATGA	GTTGCTGCTGGTTGGAGTTG
Cyr61	CCAGTGTACAGCAGCCTGAA	GGCCGGTATTTCTTCACACTC
CTGF	AGGAGTGGGTGTGTGACGAG	CGGGACAGTTGTAATGGCAG

**Table 2:** Gene set enrichment analysis.

Gene Set	Website Link
Hippo signature	http://software.broadinstitute.org/gsea/msigdb/geneset_page.jsp?geneSetName=CORDENONSI_YAP_CONSERVED_SIGNATURE&keywords=hippo
Positive regulation of cell proliferation (GO/0008284)	http://software.broadinstitute.org/gsea/msigdb/cards/GO_POSITIVE_REGULATION_OF_CELL_PROLIFERATION
GO positive regulation of cell cycle G1-S phase transition	https://www.gsea-msigdb.org/gsea/msigdb/cards/GO_POSITIVE_REGULATION_OF_CELL_CYCLE_G1_S_PHASE_TRANSITION.html

## Supplementary Materials

The following are available online at https://www.mdpi.com/2072-6694/12/12/3847/s1, Figure S1: Elevation of Hippo gene expression in OS patients. Figure S2: Role of TEAD in YAP-driven TEAD transcriptional activity. Figure S3: Role of TEAD in YAP-driven genes expression. Figure S4: Verteporfin and CA3 inhibit YAP expression and YAP-driven TEAD transcriptional activity. Figure S5: Verteporfin and CA3 stimulate OS cell apoptosis. Figure S6: Uncropped Western Blotting figures.
