# Supplementary material for: The YAP/TEAD Axis as a New Therapeutic Target in Osteosarcoma: Effect of Verteporfin and CA3 on Primary Tumor Growth
Source: Cancers (Basel). 2020 Dec 20;12(12):3847. doi: 10.3390/cancers12123847 (PMC7766439; doi:10.3390/cancers12123847)
Supplement: Supplementary file 1 [file cancers-12-03847-s001.zip › cancers-1024990-supplement.pdf]

cancers

MDPI

Supplementary Materials

The YAP/TEAD axis as a new therapeutic target in the primary growth of osteosarcoma: effect of verteporfin and CA3?

Sarah Morice, Mathilde Mullard, Regis Brion, Maryne Dupuy, Sarah Renault, Robel Tesfaye, Benjamin Ory, Bénédicte Brounais-Le Royer, Françoise Redini and Franck Verrecchia

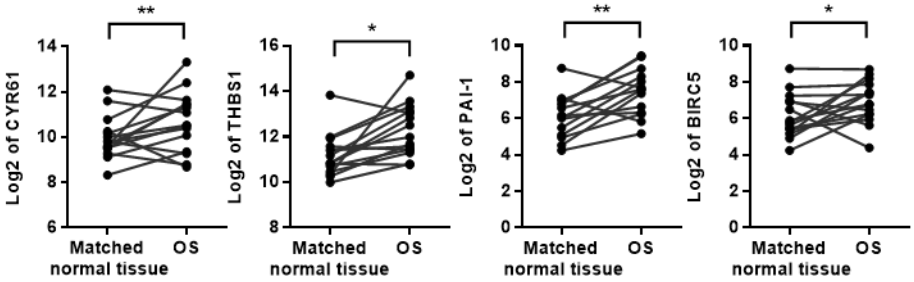

Figure S1: Elevation of Hippo gene expression in OS patients. Relative CYR61, THBS1, PAI-1 and BIRC5 genes expression in OS patient and control samples following bioinformatics analysis of RNAseq data GSE99671 [27].

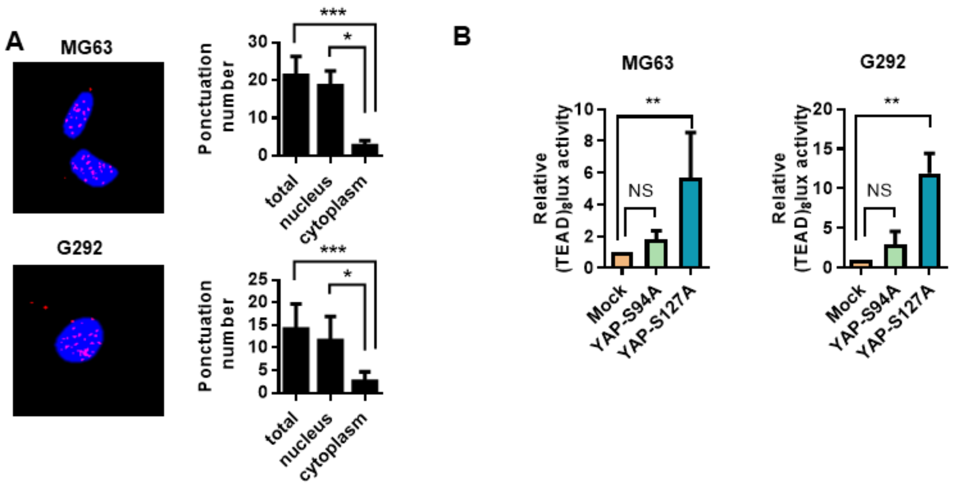

Figure S2: Role of TEAD in YAP-driven TEAD transcriptional activity. A) Localization of endogenous YAP/TEAD1 complexes by in situ PLA in MG63 and G292 OS cells. The red signal was obtained using Alexa555-labeled hybridization oligo nucleotides targeting amplified in situ PLA products. DAPI (blue) staining was used for nuclear visualization. Bars indicate means ± S.D. of three independent experiments (*p< 0.05, ***p< 0.001). B) MG63 or G292 cells were co-transfected with the TEAD-specific construct (TEAD)8-lux with or without empty, YAPS94A and YAPS127A expression vectors. Bars indicate means ± SD of four independent experiments, each performed in triplicate (**p<0.01).

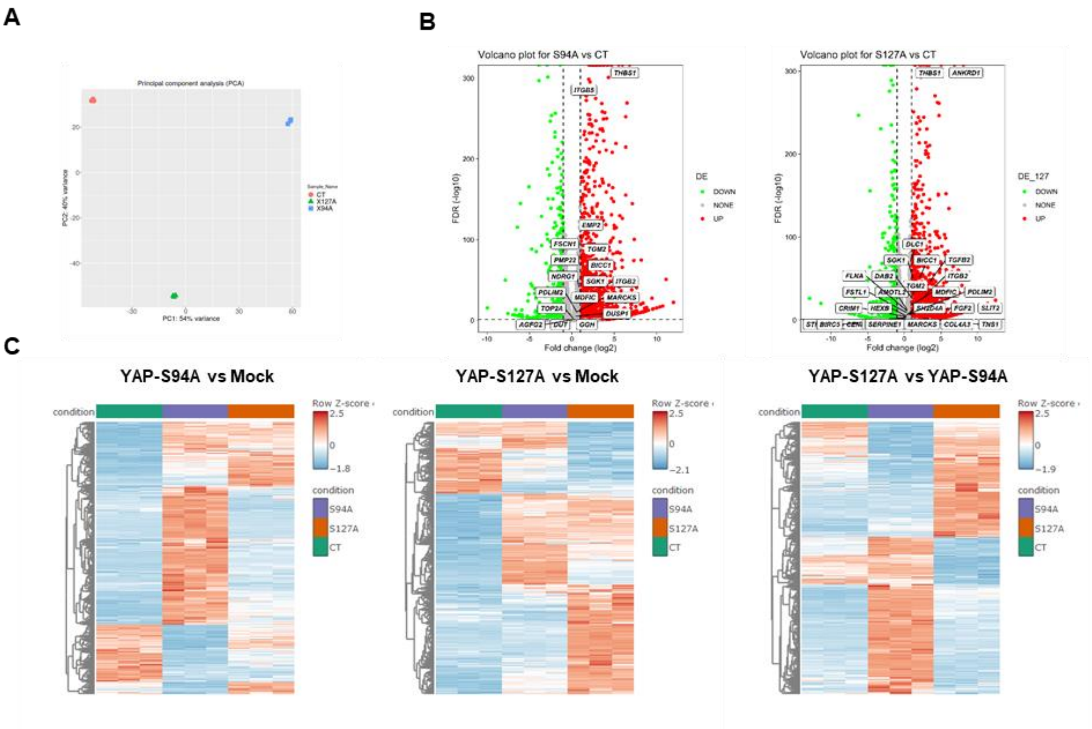

Figure S3: Role of TEAD in YAP-driven genes expression. A) PCA analysis was done to evaluate data reproducibility among replicates and to summarize genome-wide transcriptional effect of transfections. B) Volcano plot of genes differentially expressed (FDR < 0.05) in YAPS94A- vs mock-, and YAPS127A- vs mock-transfected cells. C) Heatmap of all the significantly differentially expressed genes is plotted (YAPS94A- vs mock-transfected cells, YAPS127A- vs mock-transfected cells and YAPS127A- vs mock-transfected cells). Colour scales are based on Z-scores.

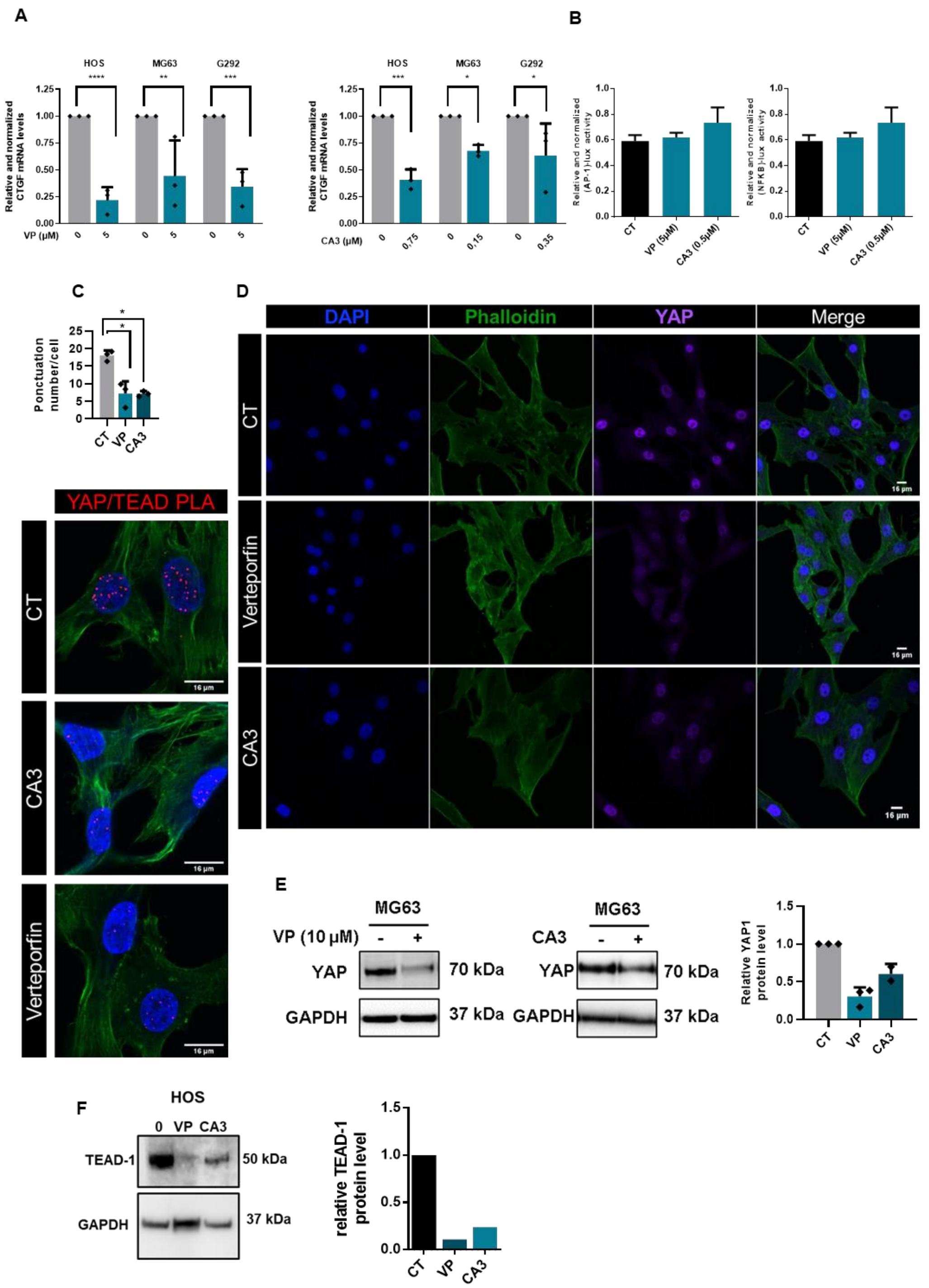

Figure S4: Verteporfin and CA3 inhibit YAP expression and YAP-driven TEAD transcriptional activity. A) CTGF mRNA steady-state levels were quantified by RT-q-PCR analysis in the presence or absence of verteporfin or CA3 48 h. Bars indicate the means ± SD of three independent experiments, each performed in triplicate (*p<0.05, **p<0.01, ***P < 0.001, **** < p 0.0001). B) HOS cells were transfected with the AP1- and NFkB-specific construct AP1-lux and NFkB-lux, respectively. 24 h after transfection, cells were treated with verteporfin or CA3 as indicated concentration for 48 h. Bars indicate means ± S.D. of an each performed in triplicate. C) Localization of YAP/TEAD1 complexes by in situ PLA experiments in MG63 cells treated or not with 10 µM verteporfin and 0.1 µM CA3 during 48 h. The red signal was obtained using Alexa555-labeled hybridization oligo nucleotides targeting amplified in situ PLA products. DAPI (blue) staining was used for nuclear visualization (lower panel). Bars indicate means ± S.D. of three independent experiments (*p<0.05, upper panel). D) MG63 were stimulate or not with 10 µM verteporfin and 0.75 µM CA3 during 48 h and were then fixed, permeabilized and stained with a monoclonal antibody directed against

YAP (far-red). F-actin cytoskeleton and nuclei were respectively revealed by phalloidine (green) and DAPI labelling (blue). Photographs representative of two independent experiments are shown. E) YAP production was detected by Western blot analysis in MG63 cells treated or not with 10 µM verteporfin and 0.6 µM CA3 during 72 h. Results shown are representative of three independent experiments (right panel). F) TEAD production was detected by Western blot analysis in HOS cells treated or not with 10 µM verteporfin and 0.6 µM CA3 during 72 h. Results shown one experiment.

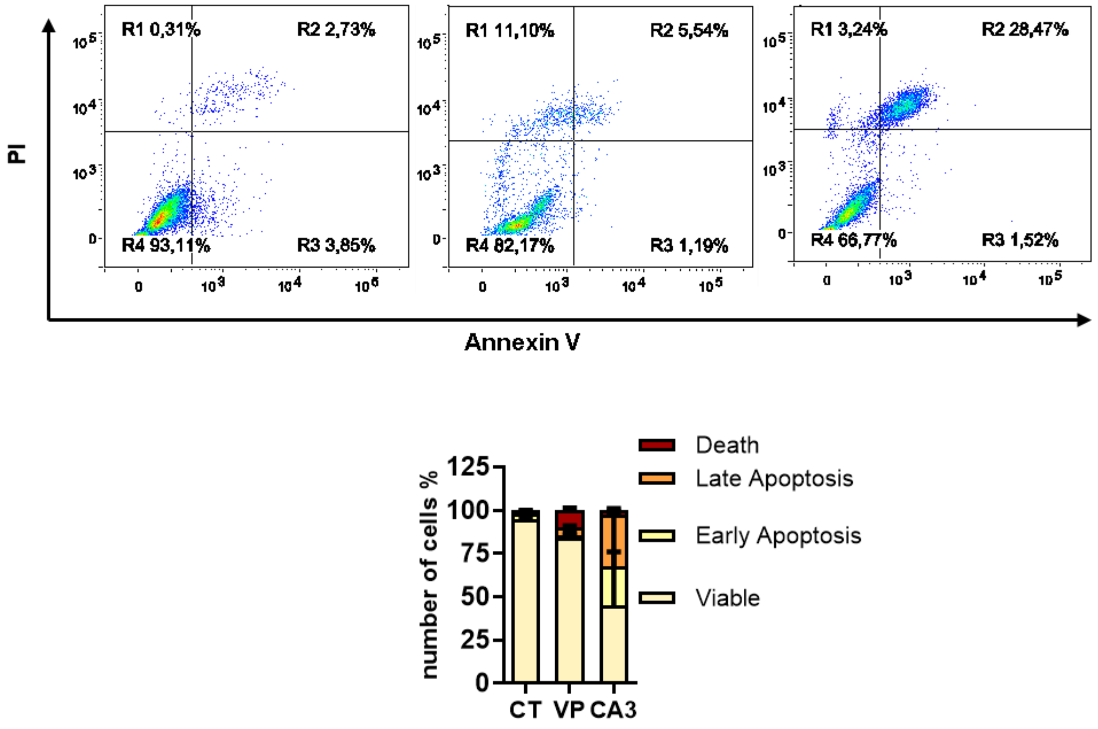

Figure S5: Verteporfin and CA3 stimulate OS cell apoptosis. Upper panels: Representative dot plots of HOS cells untreated or treated with 10 µM verteporfin or 0.75 µM CA3 for 72 h are shown (representative graphs of two experiments). Lower panels: Bars indicate the means ± SD of the relative number of lives cells, death cells, and cells in early- or late-phase apoptosis.
