# Supplementary material for: The YAP/TEAD Axis as a New Therapeutic Target in Osteosarcoma: Effect of Verteporfin and CA3 on Primary Tumor Growth
Source: Cancers (Basel). 2020 Dec 20;12(12):3847. doi: 10.3390/cancers12123847 (PMC7766439; doi:10.3390/cancers12123847)
Supplement: Supplementary file 1 [file cancers-12-03847-s001.zip › Figure S6-western blot-Full_uncut_gels.pdf]

Figure 2B

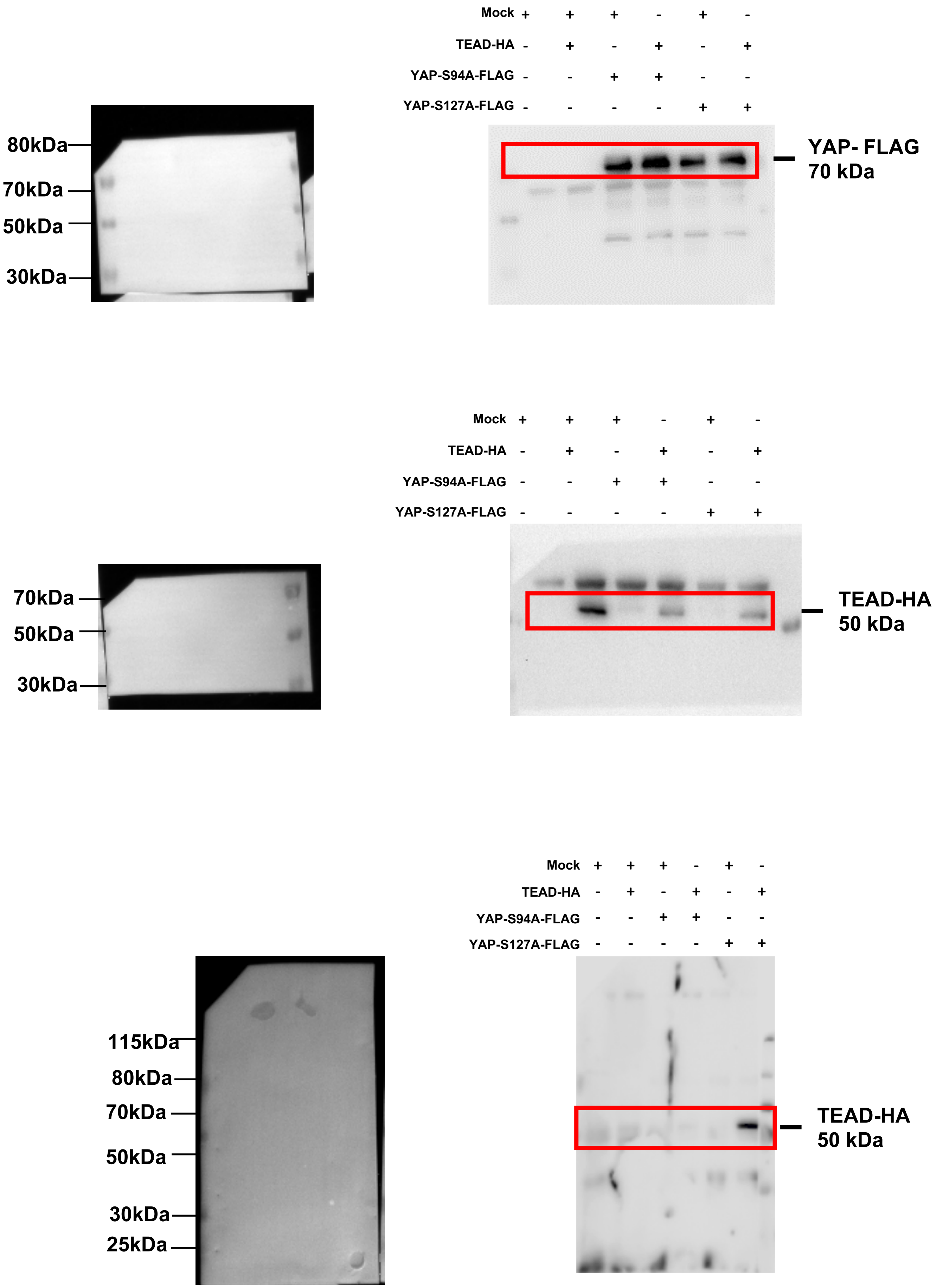

Mock + + + - + -
TEAD-HA - + - + - +
YAP-S94A-FLAG - - + + - -
YAP-S127A-FLAG - - - - + +
80kDa
70kDa
50kDa
30kDa
YAP- FLAG
70 kDa
Mock + + + - + -
TEAD-HA - + - + - +
YAP-S94A-FLAG - - + + - -
YAP-S127A-FLAG - - - - + +
70kDa
50kDa
30kDa
TEAD-HA
50 kDa
Mock + + + - + -
TEAD-HA - + - + - +
YAP-S94A-FLAG - - + + - -
YAP-S127A-FLAG - - - - + +
115kDa
80kDa
70kDa
50kDa
30kDa
25kDa
TEAD-HA
50 kDa

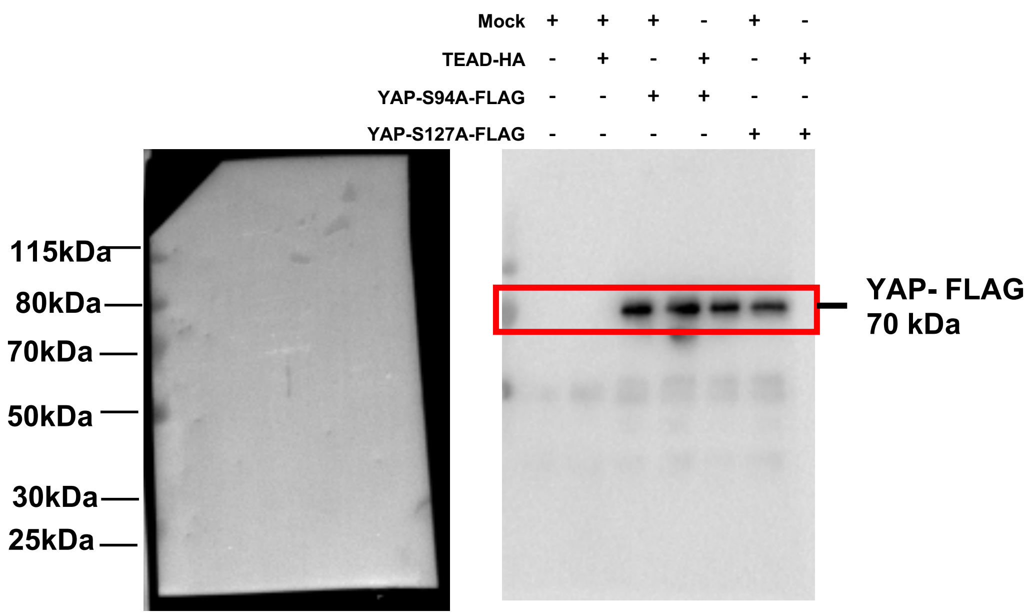

Mock + + + - + -
TEAD-HA - + - + - +
YAP-S94A-FLAG - - + + - -
YAP-S127A-FLAG - - - - + +
115kDa
80kDa
70kDa
50kDa
30kDa
25kDa
YAP- FLAG
70 kDa

Figure 2C

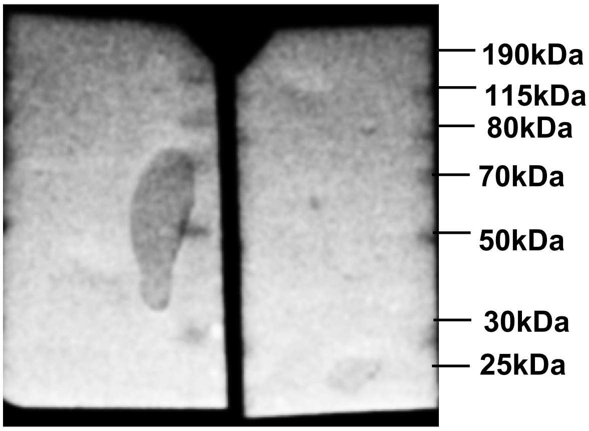
190kDa
115kDa
80kDa
70kDa
50kDa
30kDa
25kDa

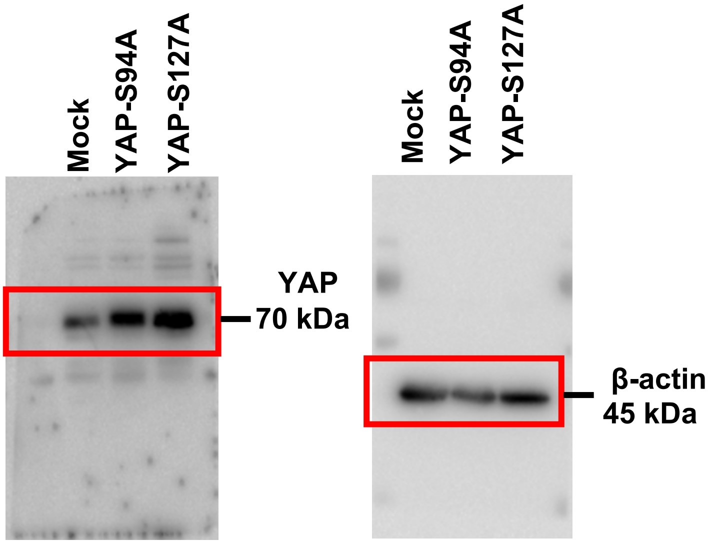
Mock
YAP-S94A
YAP-S127A
YAP
70 kDa
Mock
YAP-S94A
YAP-S127A
β-actin
45 kDa

Figure 5E

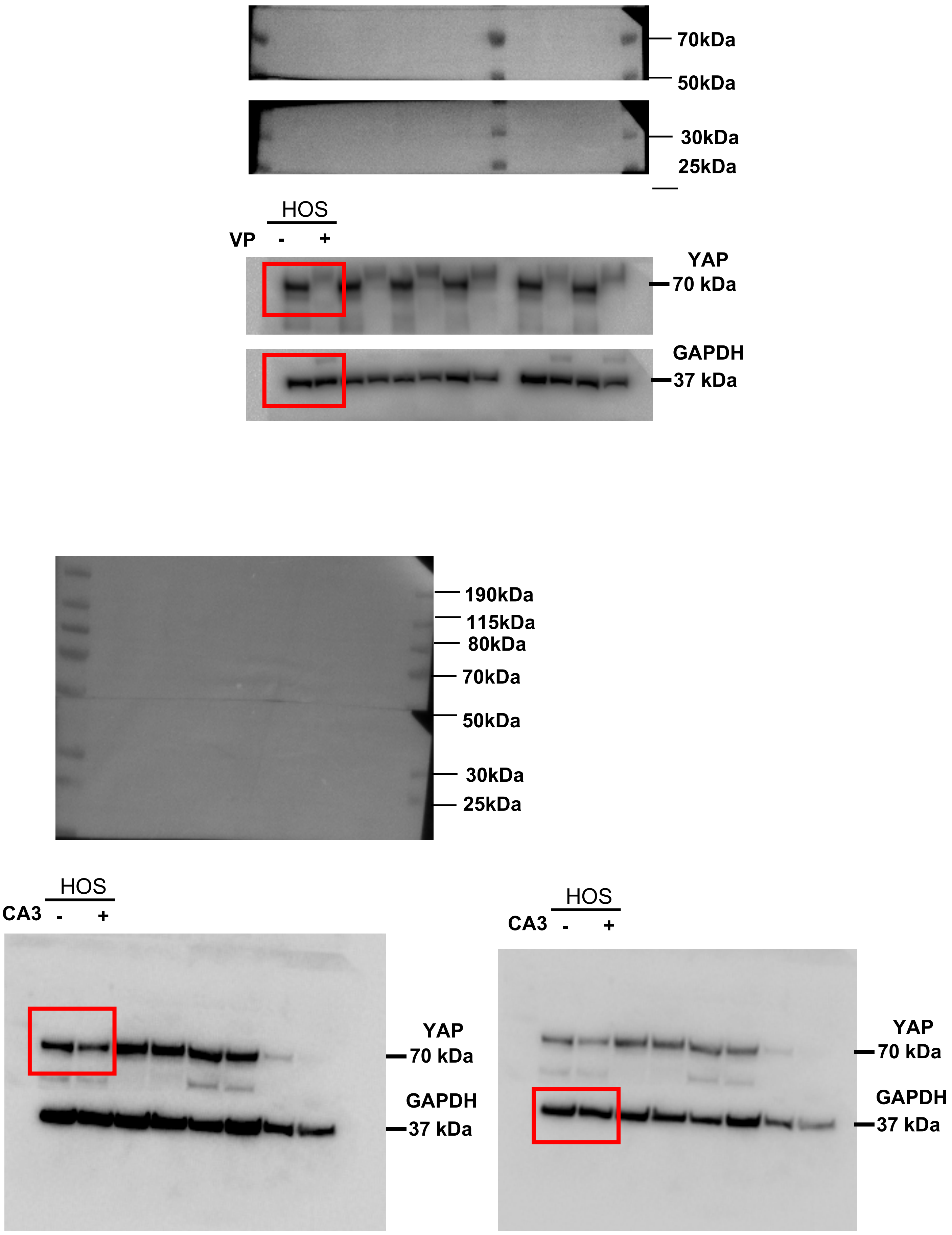
70kDa
50kDa
30kDa
25kDa
HOS
VP
-
+
YAP
70 kDa
GAPDH
37 kDa
190kDa
115kDa
80kDa
70kDa
50kDa
30kDa
25kDa
HOS
CA3
-
+
YAP
70 kDa
GAPDH
37 kDa
HOS
CA3
-
+
YAP
70 kDa
GAPDH
37 kDa

Figure S4D

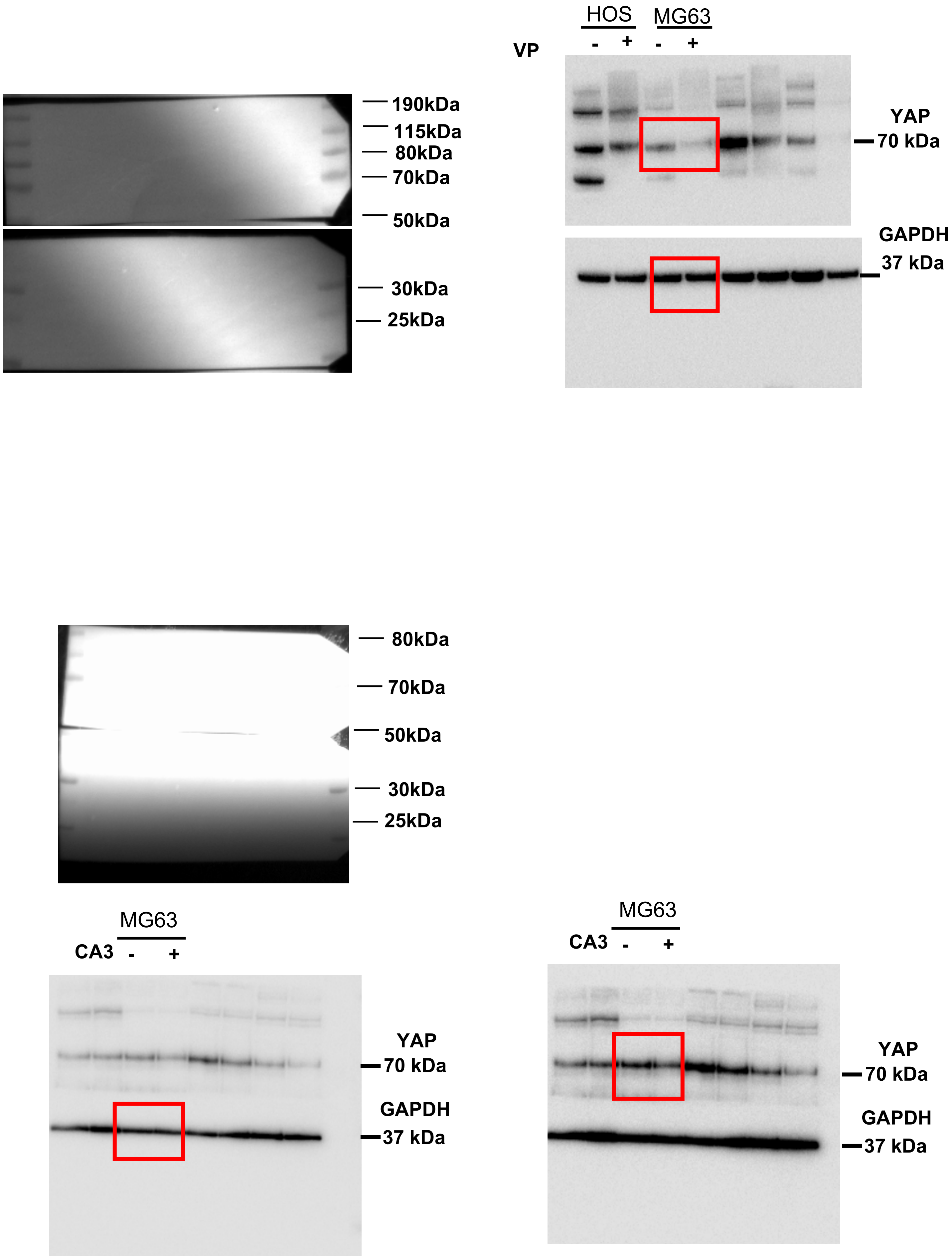
HOS
MG63
VP
- + - +
190kDa
115kDa
80kDa
70kDa
50kDa
30kDa
25kDa
YAP
70 kDa
GAPDH
37 kDa
80kDa
70kDa
50kDa
30kDa
25kDa
MG63
CA3
- +
YAP
70 kDa
GAPDH
37 kDa
MG63
CA3
- +
YAP
70 kDa
GAPDH
37 kDa
